# Post-tubercular Unilateral Lung Destruction: A Complicated Case

**DOI:** 10.7759/cureus.40035

**Published:** 2023-06-06

**Authors:** Gaurang M Aurangabadkar, Sumer S Choudhary, Shafee M Khan, Ulhas S Jadhav, Pankaj B Wagh, Saood N Ali

**Affiliations:** 1 Respiratory Medicine, Datta Meghe Medical College, Datta Meghe Institute of Higher Education and Research (Deemed University), Nagpur, IND; 2 Respiratory Medicine, Jawaharlal Nehru Medical College, Datta Meghe Institute of Higher Education and Research (Deemed University), Wardha, IND; 3 Respiratory Medicine, NKP Salve Institute of Medical Sciences and Lata Mangeshkar Hospital, Nagpur, IND

**Keywords:** two-dimensional echocardiography, mediastinal shifting, destroyed lung, pulmonary hypertension, pulmonary tuberculosis

## Abstract

Pulmonary tuberculosis is associated with long-term complications that affect both the respiratory and cardiovascular systems. We present the case of a 65-year-old male patient who presented with chief complaints of productive cough and breathlessness for the last four years. Further radiological investigations revealed a left-sided destroyed lung with left lung collapse and deviation of the mediastinum towards the left side. The patient responded well to treatment with broad-spectrum antimicrobial drugs and mucolytics.

## Introduction

Tuberculosis of the lung is considered globally to be one of the most lethal and deadly communicable diseases, especially in developing countries [1].

Pulmonary tuberculosis is an infectious disease caused by *Mycobacterium tuberculosis*. Tuberculosis of the lung is associated with a wide spectrum of chronic complications and sequelae that can significantly increase the morbidity of the affected patient [2]. One of the most important and disabling complications of pulmonary tuberculosis has been found to be the unilateral destruction of the lung parenchyma [2].

Unilateral lung destruction is considered to be one of the late complications of pulmonary tuberculosis, which usually requires years to manifest clinically [2]. This lung destruction often leads to herniation of the contralateral lung towards the affected side with an ipsilateral shifting of the mediastinum [1]. The clinical signs of lung volume loss such as shoulder drooping, undue prominence of the clavicular head of the sternocleidomastoid muscle of the neck also known as the Trail’s sign, and reduced spino-scapular distance on the affected side, can be elicited in such patients [3].

## Case presentation

A 65-year-old male patient was presented to the Respiratory Medicine outpatient department (OPD) with chief complaints of productive cough and dyspnea which was present intermittently for the last 3 years. The patient also complained of left-sided chest pain on deep inspiration and coughing. A detailed history taking of the patient revealed that the patient had a history of smear-positive pulmonary tuberculosis (TB) 2 years back for which he had taken the full course of anti-TB treatment for a period of 6 months consisting of four drugs, namely isoniazid, rifampicin, pyrazinamide, and ethambutol.

**Figure 1:**
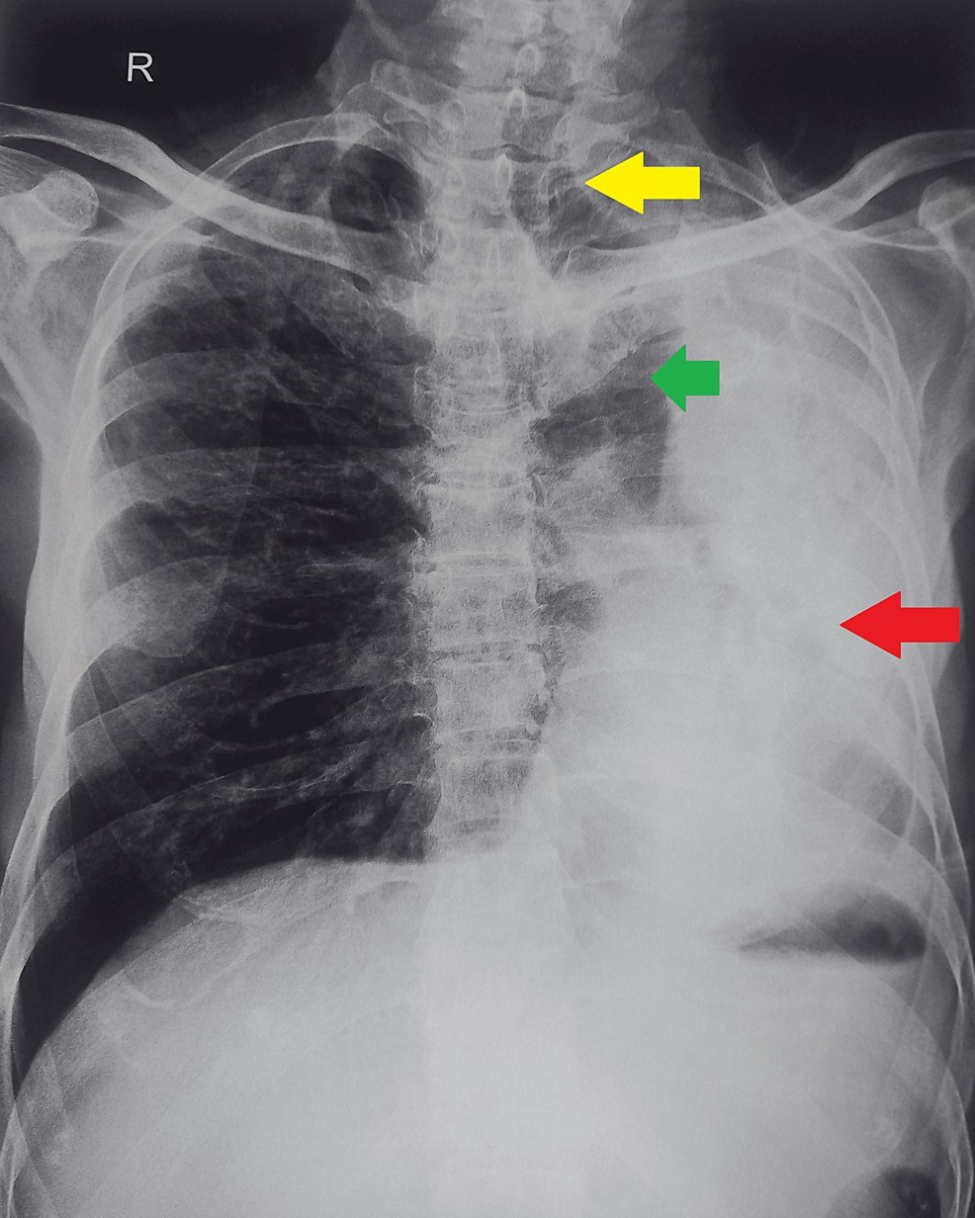
A chest X-ray posteroanterior (PA) view on admission showing the destruction of the left lung (red arrow), with ipsilateral tracheal and mediastinal shifting (yellow arrow) and herniation of a part of the right lung into the left hemithorax (green arrow).

For further evaluation of the patient, a chest X-ray posteroanterior (PA) view was done which revealed destruction of the left lung parenchyma with ipsilateral mediastinal shift and herniation of a part of the right lung into the left hemithorax (Figure 1).

**Figure 2:**
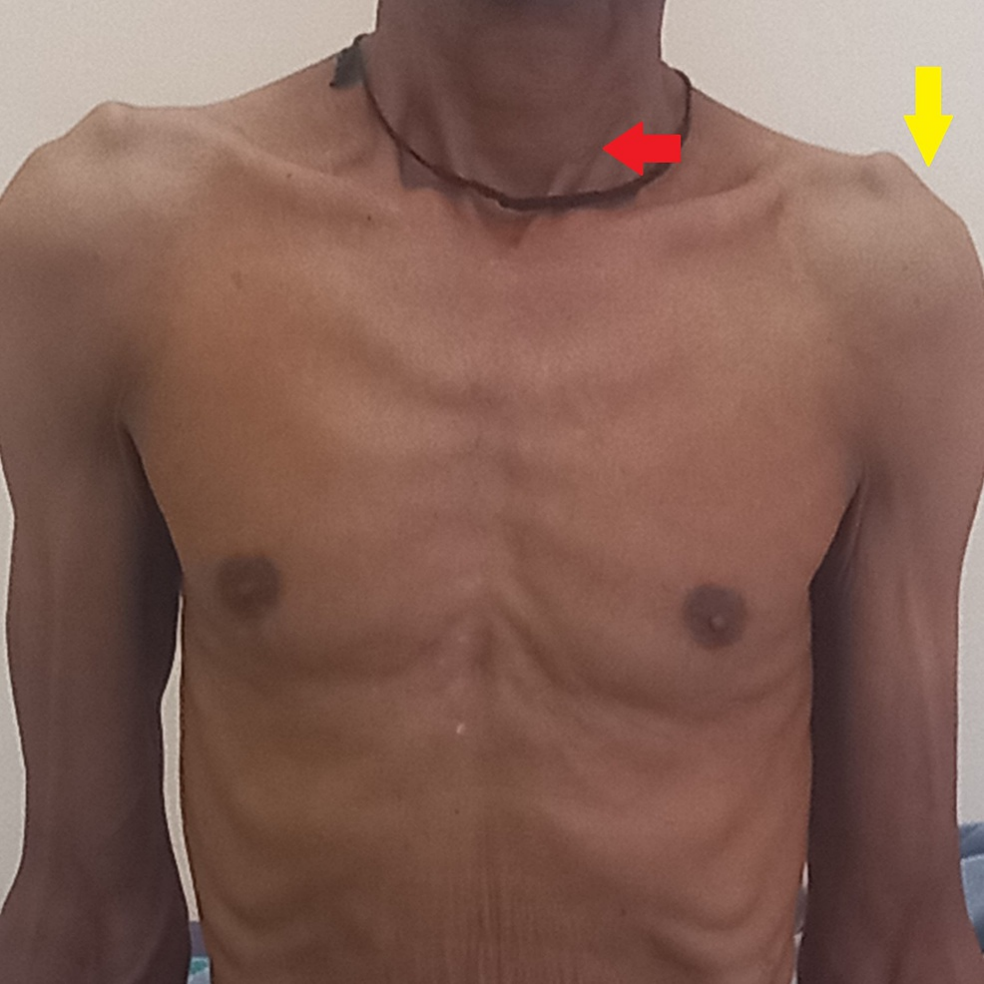
A clinical image of the chest wall and neck region of the patient showing a positive Trail’s sign on the left side (red arrow) and drooping of the left shoulder (yellow arrow).

A detailed clinical evaluation was done which revealed signs of volume loss visible in the left hemithorax, such as a positive Trail’s sign on the left side (undue prominence of the clavicular head of the sternocleidomastoid on the affected side), drooping of the left shoulder as compared to the right, evidence of rib-crowding on the left hemithorax, and a reduction in the spino-scapular distance on the left side (Figure 2).

Routine blood investigations of the patient were done which revealed no significant abnormalities. Sputum samples were sent for acid-fast bacilli (AFB) staining which was found to be negative.

The patient was started on broad-spectrum antimicrobial therapy with the addition of metronidazole for coverage against anaerobic microorganisms. The patient reported symptomatic improvement after a period of 7 days and was discharged with advice to review in the OPD after 15 days.

## Discussion

The most commonly affected parts of the lungs that are involved in a patient with pulmonary tuberculosis have been found to be the upper lobes, particularly the apical and posterior segments, with an almost identical incidence bilaterally [4]. Various studies have demonstrated that unilateral lung destruction in patients with post-primary or primary TB is most frequently seen to be involving the left lung, similar to the phase of fibrosis in cases of endobronchial tuberculosis [4,5].

This higher incidence of involvement of the left lung can be explained by the anatomical features of the left main bronchus, which is considerably smaller in diameter and longer in length as compared to the right main bronchus [6]. Due to these anatomical factors, a higher incidence of the collapse of the bronchus is noted due to lymph node swelling in the close vicinity of the bronchus. In addition to this, impaired drainage of secretions can also occur on the left side as a result of the left main bronchus having a considerably horizontal course [6,7]. Tuberculosis is associated with a variety of thoracic complications and sequelae summarized as follows (Table 1).

**Table 1:** Summary of the various complications and sequelae associated with tuberculosis [7]

Lung structures affected	Complications
Parenchymal complications	Lung destruction and cicatrization
	Aspergilloma
	Bronchogenic carcinoma
Airway complications	Bronchiectasis
	Broncho-lithiasis
	Broncho-stenosis
	Bronchiolitis obliterans
Pleural complications	Empyema thoracis
	Bronchopleural fistula
Vascular complications	Rasmussen’s aneurysm
	Pulmonary hypertension
	Pulmonary arteritis
	Bronchial arteritis
Mediastinal complications	Esophago-mediastinal fistula
	Esophago-bronchial fistula
	Constrictive pericarditis
	Fibrosing mediastinitis
Chest wall complications	Tuberculous spondylitis
	Rib tuberculosis
	Empyema necessitans

These various complications and sequelae associated with pulmonary tuberculosis as summarized above greatly contribute to the increased morbidity and mortality in such patients.

## Conclusions

Tuberculosis is a disease that affects the quality of the patient, in both the short- and the long-term, significantly increasing morbidity and mortality through the development of various thoracic complications and sequelae. Patients who develop complications such as unilateral lung parenchymal destruction post-tubercular infection usually experience frequent respiratory tract infections and persisting dyspnea due to derangement of the normal pulmonary parenchyma structures. Such patients need early identification by means of screening with chest radiographs and should be educated regarding various preventive measures such as seasonal pneumococcal and influenza vaccination as well as the institution of a pulmonary rehabilitation program to improve the quality of life and exercise capacity of the patient.
